# Diaqua­(2,6-dioxo-1,2,3,6-tetra­hydro­pyrimidin-3-ide-4-carboxyl­ato-κ^2^ *N* ^3^,*O* ^4^)(1,10-phenanthroline-κ^2^ *N*,*N*′)manganese(II)

**DOI:** 10.1107/S1600536808005230

**Published:** 2008-02-29

**Authors:** Rentao Wu, Yanmin Huo, Jikun Li, Zebao Zheng

**Affiliations:** a Department of Chemistry, Taishan University, 271021 Tai’an, Shandong, People’s Republic of China; b Shan Dong Institute of Supervision & Inspection of Product Quality, 250100 Ji’nan, Shandong, People’s Republic of China; c Department of Materials and Chemical Engineering, Taishan University, 271021 Tai’an, Shandong, People’s Republic of China

## Abstract

The title compound, [Mn(C_5_H_2_N_2_O_4_)(C_12_H_8_N_2_)(H_2_O)_2_], was synthesized by the reaction of manganese(II) acetate and orotic acid in the presence of 1,10-phenanthroline. The crystal structure exhibits inter­molecular N—H⋯O and O—H⋯O hydrogen bonds . The Mn coordination environment consists of an N_3_O_3_ donor set in an octa­hedral geometry.

## Related literature

For biochemical processes, see: Mukhopadhyay *et al.*, (2004 ▶); Ren *et al.*, (2005 ▶). For bioinorganic and pharmaceutical studies, see: Lieberman *et al.*, (1955 ▶). For coordination chemistry and other aspects, see: Darensbourg *et al.*, (1998 ▶). For complexes of the orotate ligand, see: Hambley *et al.*, (1995 ▶); Nepveu *et al.*, (1995 ▶).
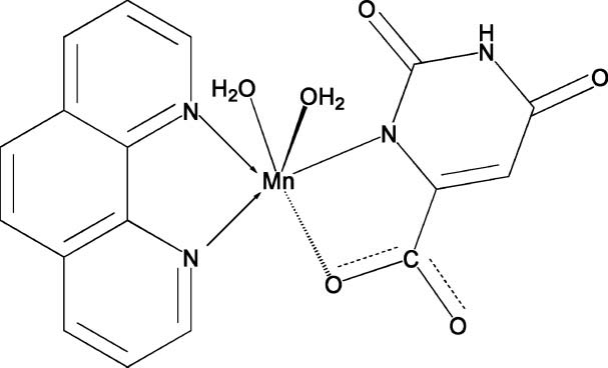


## Experimental

### 

#### Crystal data

[Mn(C_5_H_2_N_2_O_4_)(C_12_H_8_N_2_)(H_2_O)_2_]
*M* *_r_* = 425.26
Triclinic, 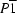

*a* = 8.3173 (2) Å
*b* = 8.9875 (2) Å
*c* = 11.9509 (3) Å
α = 78.2780 (10)°
β = 82.9100 (10)°
γ = 74.7440 (10)°
*V* = 841.58 (3) Å^3^
*Z* = 2
Mo *K*α radiation
μ = 0.83 mm^−1^
*T* = 293 (2) K
0.15 × 0.12 × 0.10 mm

#### Data collection

Bruker SMART diffractometer
Absorption correction: multi-scan (*SADABS*; Sheldrick, 1996 ▶) *T*_min_ = 0.885, *T*_max_ = 0.921
9718 measured reflections
2950 independent reflections
2539 reflections with *I* > 2σ(*I*)
*R*_int_ = 0.027

#### Refinement

*R*[*F* ^2^ > 2σ(*F* ^2^)] = 0.030
*wR*(*F* ^2^) = 0.072
*S* = 1.05
2950 reflections
257 parameters
H-atom parameters constrained
Δρ_max_ = 0.21 e Å^−3^
Δρ_min_ = −0.22 e Å^−3^

### 

Data collection: *SMART* (Bruker, 2007 ▶); cell refinement: *SAINT* (Bruker, 2007 ▶); data reduction: *SAINT*; program(s) used to solve structure: *SHELXS97* (Sheldrick, 2008 ▶); program(s) used to refine structure: *SHELXL97* (Sheldrick, 2008 ▶); molecular graphics: *SHELXTL* (Sheldrick, 2008 ▶); software used to prepare material for publication: *SHELXL97*.

## Supplementary Material

Crystal structure: contains datablocks I, global. DOI: 10.1107/S1600536808005230/om2206sup1.cif

Structure factors: contains datablocks I. DOI: 10.1107/S1600536808005230/om2206Isup2.hkl

Additional supplementary materials:  crystallographic information; 3D view; checkCIF report

## Figures and Tables

**Table 1:** Selected bond lengths (Å)

Mn1—O1	2.1231 (15)
Mn1—O6	2.1439 (15)
Mn1—O5	2.1852 (16)
Mn1—N1	2.2686 (16)
Mn1—N3	2.2880 (18)
Mn1—N4	2.2979 (19)

**Table 2:** Hydrogen-bond geometry (Å, °)

*D*—H⋯*A*	*D*—H	H⋯*A*	*D*⋯*A*	*D*—H⋯*A*
N2—H2⋯O4^i^	0.86	1.97	2.831 (2)	177
O5—H5*A*⋯O4^ii^	0.85	2.00	2.829 (2)	164
O5—H5*B*⋯O3^iii^	0.85	1.95	2.769 (2)	162
O6—H6*A*⋯O2^iv^	0.85	1.79	2.627 (2)	170
O6—H6*B*⋯O3	0.85	1.92	2.675 (2)	147

Symmetry codes: (i) 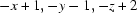; (ii) 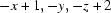; (iii) 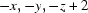; (iv) 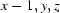.

**Fig. 1.**
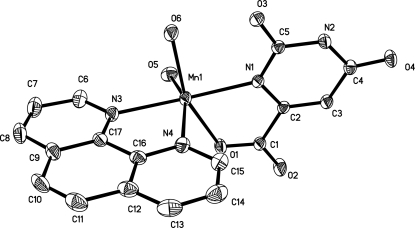
The structure of the title compound, showing 30% probability displacement ellipsoids and the atom-numbering scheme. The H atoms are omitted.

## supplementary crystallographic information

### Comment

Manganese is an important element in organisms and involved in many biochemical processes (Mukhopadhyay *et al.*, 2004 and Ren *et al.*, 2005). It may be observed in the active parts of many enzymes. Orotic acid (2, 6-dioxo-1, 2, 3, 6-tetrahydropyrimidine-4- carboxylic acid), an important pyrimidine derivative as the effective precursor in the biosynthesis of pyrimidine base of nucleic acids in living organisms, plays an important role in bioinorganic chemistry and pharmaceutical studies (Lieberman *et al.*, 1955), material science, coordination chemistry and other aspects (Darensbourg *et al.*, 1998). Many complexes of the orotate ligand have been reported (Hambley *et al.*, 1995).

In the title compound (Fig, 1), Mn(C_12_H_8_N_2_)(C_5_H_2_N_2_O_4_)(H_2_O)_2_, the Mn(II) ion is coordinated by N and O atoms from the orotate (2, 6-dioxo-1, 2, 3, 6-tetrahydropyrimidine- 4-carboxylate) ligand. The bond lengths and angles are in good agreement with reported values (Nepveu *et al.*, 1995). In the crystal structure, the molecule with its two coordinated water molecules is linked into infinite chains by O—H···O and N—H···O hydrogen bonds.

### Experimental

Orotic acid (0.0012 mol), 1, 10-phenanthroline (0.0012 mol) and Mn(CH_3_COO)_2_.2H_2_O (0.0012 mol, 0.294 g) in 120 ml of water were stirred at 373 K for 24 h; the pH of the solution was adjusted to 6 using a dilute aqueous solution of ammonia. After evaporation of the solution for two weeks, colorless block-like crystals were isolated by filtration. Analysis, calculated for C_17_H_14_N_4_O_6_Mn: C 48.01, H 3.32, N 13.17; found: C 48.00, H 3.30, N 13.16. Crystals suitable for single-crystal X-ray analysis were selected directly from the sample.

### Refinement

All H atoms were initially located in a difference Fourier map, but placed in idealized positions (C—H 0.93 Å, N—H 0.86 Å), with *U*_iso_(H) = 1.2*U*_eq_(C). The O—H bond lengths were constrained to 0.85Å and the isotropic thermal parameters of the H atoms bonded to water were refined.

### Crystal data

**Table:**

[Mn(C_5_H_2_N_2_O_4_)(C_12_H_8_N_2_)(H_2_O)_2_]	*Z* = 2
*M**_r_* = 425.26	*F*_000_ = 434
Triclinic, *P*1	*D*_x_ = 1.678 Mg m^−^^3^
*a* = 8.3173 (2) Å	Mo *K*α radiation λ = 0.71073 Å
*b* = 8.9875 (2) Å	Cell parameters from 3161 reflections
*c* = 11.9509 (3) Å	θ = 2.4–24.5º
α = 78.2780 (10)º	µ = 0.83 mm^−^^1^
β = 82.9100 (10)º	*T* = 293 (2) K
γ = 74.7440 (10)º	Block, colorless
*V* = 841.58 (3) Å^3^	0.15 × 0.12 × 0.10 mm

### Data collection

**Table:**

Bruker SMART diffractometer	2950 independent reflections
Radiation source: fine-focus sealed tube	2539 reflections with *I* > 2σ(*I*)
Monochromator: graphite	*R*_int_ = 0.027
*T* = 293(2) K	θ_max_ = 25.0º
φ and ω scans	θ_min_ = 1.8º
Absorption correction: multi-scan(SADABS; Sheldrick, 1996)	*h* = −9→9
*T*_min_ = 0.886, *T*_max_ = 0.922	*k* = −10→10
9718 measured reflections	*l* = −14→13

### Refinement

**Table:**

Refinement on *F*^2^	Secondary atom site location: difference Fourier map
Least-squares matrix: full	Hydrogen site location: inferred from neighbouring sites
*R*[*F*^2^ > 2σ(*F*^2^)] = 0.030	H-atom parameters constrained
*wR*(*F*^2^) = 0.072	*w* = 1/[σ^2^(*F*_o_^2^) + (0.0269*P*)^2^ + 0.4894*P*] where *P* = (*F*_o_^2^ + 2*F*_c_^2^)/3
*S* = 1.05	(Δ/σ)_max_ = 0.001
2950 reflections	Δρ_max_ = 0.22 e Å^−^^3^
257 parameters	Δρ_min_ = −0.22 e Å^−^^3^
Primary atom site location: structure-invariant direct methods	Extinction correction: none

### Special details

**Table:**

Geometry. All e.s.d.'s (except the e.s.d. in the dihedral angle between two l.s. planes) are estimated using the full covariance matrix. The cell e.s.d.'s are taken into account individually in the estimation of e.s.d.'s in distances, angles and torsion angles; correlations between e.s.d.'s in cell parameters are only used when they are defined by crystal symmetry. An approximate (isotropic) treatment of cell e.s.d.'s is used for estimating e.s.d.'s involving l.s. planes.
Refinement. Refinement of *F*^2^ against ALL reflections. The weighted *R*-factor *wR* and goodness of fit *S* are based on *F*^2^, conventional *R*-factors *R* are based on *F*, with *F* set to zero for negative *F*^2^. The threshold expression of *F*^2^ > σ(*F*^2^) is used only for calculating *R*-factors(gt) *etc*. and is not relevant to the choice of reflections for refinement. *R*-factors based on *F*^2^ are statistically about twice as large as those based on *F*, and *R*- factors based on ALL data will be even larger.

### Fractional atomic coordinates and isotropic or equivalent isotropic displacement parameters (Å^2^)

**Table:**

	*x*	*y*	*z*	*U*_iso_*/*U*_eq_	
Mn1	0.11775 (4)	0.19441 (4)	0.78756 (3)	0.03047 (11)	
N1	0.2989 (2)	−0.03517 (19)	0.85359 (15)	0.0276 (4)	
N2	0.3993 (2)	−0.29802 (19)	0.93896 (15)	0.0305 (4)	
H2	0.3786	−0.3884	0.9636	0.037*	
N3	−0.0891 (2)	0.3718 (2)	0.68697 (16)	0.0336 (4)	
N4	0.1453 (2)	0.1395 (2)	0.60556 (16)	0.0358 (4)	
O1	0.35761 (18)	0.24240 (17)	0.75978 (15)	0.0416 (4)	
O2	0.62451 (19)	0.16456 (19)	0.79231 (17)	0.0532 (5)	
O3	0.13026 (17)	−0.20586 (16)	0.89654 (13)	0.0335 (4)	
O4	0.66615 (18)	−0.40215 (16)	0.98859 (14)	0.0385 (4)	
O5	0.0660 (2)	0.33271 (19)	0.92439 (14)	0.0414 (4)	
H5A	0.1454	0.3672	0.9392	0.085 (11)*	
H5B	0.0168	0.3024	0.9883	0.084 (11)*	
O6	−0.07494 (19)	0.08117 (19)	0.86706 (15)	0.0437 (4)	
H6A	−0.1673	0.1030	0.8365	0.071 (10)*	
H6B	−0.0474	−0.0182	0.8853	0.081 (11)*	
C1	0.4836 (3)	0.1428 (2)	0.79908 (19)	0.0312 (5)	
C2	0.4575 (2)	−0.0191 (2)	0.85488 (17)	0.0258 (4)	
C3	0.5858 (3)	−0.1354 (2)	0.89906 (19)	0.0304 (5)	
H3	0.6906	−0.1167	0.8987	0.036*	
C4	0.5589 (3)	−0.2856 (2)	0.94576 (18)	0.0294 (5)	
C5	0.2696 (2)	−0.1773 (2)	0.89586 (17)	0.0266 (4)	
C6	−0.2058 (3)	0.4826 (3)	0.7282 (2)	0.0450 (6)	
H6	−0.2016	0.4959	0.8028	0.054*	
C7	−0.3352 (3)	0.5805 (3)	0.6641 (3)	0.0534 (7)	
H7	−0.4157	0.6565	0.6962	0.064*	
C8	−0.3428 (3)	0.5639 (3)	0.5547 (3)	0.0506 (7)	
H8	−0.4273	0.6299	0.5110	0.061*	
C9	−0.2226 (3)	0.4470 (3)	0.5079 (2)	0.0409 (6)	
C10	−0.2204 (3)	0.4214 (3)	0.3930 (2)	0.0508 (7)	
H10	−0.3020	0.4849	0.3458	0.061*	
C11	−0.1026 (4)	0.3072 (3)	0.3523 (2)	0.0518 (7)	
H11	−0.1045	0.2926	0.2776	0.062*	
C12	0.0259 (3)	0.2076 (3)	0.4220 (2)	0.0427 (6)	
C13	0.1512 (4)	0.0871 (3)	0.3838 (2)	0.0521 (7)	
H13	0.1534	0.0681	0.3099	0.062*	
C14	0.2700 (3)	−0.0023 (3)	0.4549 (2)	0.0525 (7)	
H14	0.3547	−0.0817	0.4300	0.063*	
C15	0.2621 (3)	0.0277 (3)	0.5655 (2)	0.0439 (6)	
H15	0.3431	−0.0342	0.6138	0.053*	
C16	0.0279 (3)	0.2301 (3)	0.53475 (19)	0.0343 (5)	
C17	−0.0985 (3)	0.3524 (2)	0.57867 (19)	0.0329 (5)	

### Atomic displacement parameters (Å^2^)

**Table:**

	*U*^11^	*U*^22^	*U*^33^	*U*^12^	*U*^13^	*U*^23^
Mn1	0.02175 (18)	0.03294 (19)	0.0356 (2)	−0.00587 (13)	−0.00776 (14)	−0.00124 (14)
N1	0.0201 (9)	0.0290 (9)	0.0327 (10)	−0.0066 (7)	−0.0040 (7)	−0.0010 (7)
N2	0.0263 (9)	0.0241 (9)	0.0415 (11)	−0.0077 (7)	−0.0084 (8)	−0.0008 (8)
N3	0.0267 (10)	0.0351 (10)	0.0387 (11)	−0.0079 (8)	−0.0068 (8)	−0.0029 (8)
N4	0.0300 (10)	0.0404 (10)	0.0362 (11)	−0.0080 (8)	−0.0044 (8)	−0.0048 (9)
O1	0.0251 (8)	0.0330 (8)	0.0610 (11)	−0.0081 (7)	−0.0124 (7)	0.0117 (7)
O2	0.0238 (9)	0.0409 (9)	0.0900 (14)	−0.0128 (7)	−0.0162 (9)	0.0134 (9)
O3	0.0240 (8)	0.0351 (8)	0.0431 (9)	−0.0116 (6)	−0.0060 (7)	−0.0026 (7)
O4	0.0276 (8)	0.0280 (8)	0.0585 (11)	−0.0045 (6)	−0.0166 (7)	0.0006 (7)
O5	0.0368 (9)	0.0515 (10)	0.0428 (10)	−0.0209 (8)	−0.0032 (8)	−0.0105 (8)
O6	0.0266 (9)	0.0398 (10)	0.0637 (12)	−0.0115 (7)	−0.0110 (8)	0.0022 (8)
C1	0.0237 (11)	0.0313 (11)	0.0374 (13)	−0.0075 (9)	−0.0050 (9)	−0.0011 (9)
C2	0.0211 (10)	0.0297 (10)	0.0268 (11)	−0.0063 (8)	−0.0028 (8)	−0.0046 (8)
C3	0.0213 (11)	0.0303 (11)	0.0401 (13)	−0.0079 (9)	−0.0066 (9)	−0.0031 (9)
C4	0.0254 (11)	0.0298 (11)	0.0338 (12)	−0.0055 (9)	−0.0067 (9)	−0.0069 (9)
C5	0.0242 (11)	0.0298 (11)	0.0265 (11)	−0.0070 (8)	−0.0030 (9)	−0.0052 (9)
C6	0.0357 (14)	0.0430 (14)	0.0547 (16)	−0.0043 (11)	−0.0076 (12)	−0.0096 (12)
C7	0.0371 (14)	0.0415 (14)	0.077 (2)	−0.0003 (11)	−0.0107 (14)	−0.0080 (14)
C8	0.0368 (14)	0.0400 (14)	0.073 (2)	−0.0092 (11)	−0.0255 (13)	0.0083 (13)
C9	0.0365 (13)	0.0380 (13)	0.0495 (15)	−0.0170 (10)	−0.0175 (11)	0.0083 (11)
C10	0.0535 (16)	0.0579 (16)	0.0433 (16)	−0.0286 (14)	−0.0259 (13)	0.0189 (13)
C11	0.0628 (18)	0.0657 (18)	0.0334 (14)	−0.0306 (15)	−0.0145 (13)	0.0025 (13)
C12	0.0473 (15)	0.0529 (15)	0.0341 (14)	−0.0268 (12)	−0.0046 (11)	−0.0022 (11)
C13	0.0637 (18)	0.0646 (17)	0.0367 (15)	−0.0308 (15)	0.0069 (13)	−0.0155 (13)
C14	0.0529 (17)	0.0530 (16)	0.0520 (17)	−0.0133 (13)	0.0118 (14)	−0.0193 (13)
C15	0.0381 (14)	0.0452 (14)	0.0460 (15)	−0.0065 (11)	−0.0009 (11)	−0.0088 (12)
C16	0.0335 (12)	0.0402 (12)	0.0323 (13)	−0.0184 (10)	−0.0046 (10)	0.0006 (10)
C17	0.0290 (12)	0.0359 (12)	0.0355 (13)	−0.0160 (9)	−0.0076 (10)	0.0034 (10)

### Geometric parameters (Å, °)

**Table:**

Mn1—O1	2.1231 (15)	C2—C3	1.356 (3)
Mn1—O6	2.1439 (15)	C3—C4	1.416 (3)
Mn1—O5	2.1852 (16)	C3—H3	0.9300
Mn1—N1	2.2686 (16)	C6—C7	1.396 (3)
Mn1—N3	2.2880 (18)	C6—H6	0.9300
Mn1—N4	2.2979 (19)	C7—C8	1.356 (4)
N1—C5	1.348 (3)	C7—H7	0.9300
N1—C2	1.366 (2)	C8—C9	1.401 (4)
N2—C4	1.375 (3)	C8—H8	0.9300
N2—C5	1.378 (3)	C9—C17	1.402 (3)
N2—H2	0.8600	C9—C10	1.435 (4)
N3—C6	1.323 (3)	C10—C11	1.344 (4)
N3—C17	1.355 (3)	C10—H10	0.9300
N4—C15	1.323 (3)	C11—C12	1.432 (4)
N4—C16	1.356 (3)	C11—H11	0.9300
O1—C1	1.256 (2)	C12—C13	1.399 (4)
O2—C1	1.229 (2)	C12—C16	1.405 (3)
O3—C5	1.250 (2)	C13—C14	1.362 (4)
O4—C4	1.248 (2)	C13—H13	0.9300
O5—H5A	0.8500	C14—C15	1.392 (4)
O5—H5B	0.8500	C14—H14	0.9300
O6—H6A	0.8499	C15—H15	0.9300
O6—H6B	0.8501	C16—C17	1.442 (3)
C1—C2	1.531 (3)		
			
O1—Mn1—O6	157.87 (6)	O4—C4—N2	119.95 (18)
O1—Mn1—O5	87.31 (6)	O4—C4—C3	125.78 (19)
O6—Mn1—O5	88.81 (6)	N2—C4—C3	114.26 (18)
O1—Mn1—N1	74.55 (6)	O3—C5—N1	123.06 (18)
O6—Mn1—N1	85.75 (6)	O3—C5—N2	118.06 (18)
O5—Mn1—N1	106.65 (6)	N1—C5—N2	118.87 (17)
O1—Mn1—N3	116.16 (6)	N3—C6—C7	122.6 (3)
O6—Mn1—N3	85.65 (6)	N3—C6—H6	118.7
O5—Mn1—N3	90.50 (6)	C7—C6—H6	118.7
N1—Mn1—N3	160.61 (6)	C8—C7—C6	119.6 (3)
O1—Mn1—N4	89.63 (7)	C8—C7—H7	120.2
O6—Mn1—N4	101.40 (7)	C6—C7—H7	120.2
O5—Mn1—N4	159.04 (7)	C7—C8—C9	119.7 (2)
N1—Mn1—N4	92.45 (6)	C7—C8—H8	120.1
N3—Mn1—N4	72.31 (7)	C9—C8—H8	120.1
C5—N1—C2	117.78 (17)	C8—C9—C17	117.0 (2)
C5—N1—Mn1	129.53 (13)	C8—C9—C10	123.6 (2)
C2—N1—Mn1	112.58 (12)	C17—C9—C10	119.4 (2)
C4—N2—C5	125.27 (17)	C11—C10—C9	121.2 (2)
C4—N2—H2	117.4	C11—C10—H10	119.4
C5—N2—H2	117.4	C9—C10—H10	119.4
C6—N3—C17	118.0 (2)	C10—C11—C12	121.0 (2)
C6—N3—Mn1	125.65 (16)	C10—C11—H11	119.5
C17—N3—Mn1	116.18 (14)	C12—C11—H11	119.5
C15—N4—C16	118.1 (2)	C13—C12—C16	117.5 (2)
C15—N4—Mn1	126.10 (16)	C13—C12—C11	123.3 (2)
C16—N4—Mn1	115.76 (15)	C16—C12—C11	119.2 (2)
C1—O1—Mn1	120.96 (13)	C14—C13—C12	120.0 (2)
Mn1—O5—H5A	117.0	C14—C13—H13	120.0
Mn1—O5—H5B	121.2	C12—C13—H13	120.0
H5A—O5—H5B	106.8	C13—C14—C15	118.7 (2)
Mn1—O6—H6A	119.7	C13—C14—H14	120.6
Mn1—O6—H6B	117.1	C15—C14—H14	120.6
H6A—O6—H6B	105.6	N4—C15—C14	123.4 (2)
O2—C1—O1	124.9 (2)	N4—C15—H15	118.3
O2—C1—C2	118.71 (18)	C14—C15—H15	118.3
O1—C1—C2	116.29 (17)	N4—C16—C12	122.2 (2)
C3—C2—N1	124.23 (19)	N4—C16—C17	117.9 (2)
C3—C2—C1	120.98 (18)	C12—C16—C17	119.9 (2)
N1—C2—C1	114.78 (17)	N3—C17—C9	123.0 (2)
C2—C3—C4	119.51 (18)	N3—C17—C16	117.71 (19)
C2—C3—H3	120.2	C9—C17—C16	119.2 (2)
C4—C3—H3	120.2		
			
O1—Mn1—N1—C5	−176.35 (19)	C5—N2—C4—O4	178.6 (2)
O6—Mn1—N1—C5	13.84 (18)	C5—N2—C4—C3	−2.7 (3)
O5—Mn1—N1—C5	101.32 (18)	C2—C3—C4—O4	179.8 (2)
N3—Mn1—N1—C5	−50.0 (3)	C2—C3—C4—N2	1.1 (3)
N4—Mn1—N1—C5	−87.41 (18)	C2—N1—C5—O3	−178.39 (19)
O1—Mn1—N1—C2	7.66 (13)	Mn1—N1—C5—O3	5.8 (3)
O6—Mn1—N1—C2	−162.15 (14)	C2—N1—C5—N2	0.6 (3)
O5—Mn1—N1—C2	−74.67 (14)	Mn1—N1—C5—N2	−175.20 (13)
N3—Mn1—N1—C2	133.99 (19)	C4—N2—C5—O3	−179.11 (19)
N4—Mn1—N1—C2	96.60 (14)	C4—N2—C5—N1	1.8 (3)
O1—Mn1—N3—C6	−101.28 (19)	C17—N3—C6—C7	−1.0 (3)
O6—Mn1—N3—C6	74.74 (19)	Mn1—N3—C6—C7	−175.60 (18)
O5—Mn1—N3—C6	−14.03 (19)	N3—C6—C7—C8	−0.6 (4)
N1—Mn1—N3—C6	138.6 (2)	C6—C7—C8—C9	1.2 (4)
N4—Mn1—N3—C6	178.2 (2)	C7—C8—C9—C17	−0.3 (3)
O1—Mn1—N3—C17	84.04 (15)	C7—C8—C9—C10	−179.7 (2)
O6—Mn1—N3—C17	−99.94 (15)	C8—C9—C10—C11	−179.8 (2)
O5—Mn1—N3—C17	171.29 (15)	C17—C9—C10—C11	0.7 (4)
N1—Mn1—N3—C17	−36.1 (3)	C9—C10—C11—C12	−0.4 (4)
N4—Mn1—N3—C17	3.49 (14)	C10—C11—C12—C13	180.0 (2)
O1—Mn1—N4—C15	60.93 (19)	C10—C11—C12—C16	0.1 (4)
O6—Mn1—N4—C15	−99.75 (19)	C16—C12—C13—C14	−0.5 (4)
O5—Mn1—N4—C15	142.4 (2)	C11—C12—C13—C14	179.6 (2)
N1—Mn1—N4—C15	−13.59 (19)	C12—C13—C14—C15	0.8 (4)
N3—Mn1—N4—C15	178.6 (2)	C16—N4—C15—C14	−0.4 (3)
O1—Mn1—N4—C16	−120.22 (15)	Mn1—N4—C15—C14	178.45 (18)
O6—Mn1—N4—C16	79.11 (15)	C13—C14—C15—N4	−0.4 (4)
O5—Mn1—N4—C16	−38.7 (3)	C15—N4—C16—C12	0.7 (3)
N1—Mn1—N4—C16	165.26 (15)	Mn1—N4—C16—C12	−178.23 (16)
N3—Mn1—N4—C16	−2.52 (14)	C15—N4—C16—C17	−179.66 (19)
O6—Mn1—O1—C1	19.9 (3)	Mn1—N4—C16—C17	1.4 (2)
O5—Mn1—O1—C1	100.05 (18)	C13—C12—C16—N4	−0.3 (3)
N1—Mn1—O1—C1	−8.05 (17)	C11—C12—C16—N4	179.6 (2)
N3—Mn1—O1—C1	−170.71 (16)	C13—C12—C16—C17	−179.9 (2)
N4—Mn1—O1—C1	−100.69 (18)	C11—C12—C16—C17	0.0 (3)
Mn1—O1—C1—O2	−175.75 (19)	C6—N3—C17—C9	2.0 (3)
Mn1—O1—C1—C2	6.9 (3)	Mn1—N3—C17—C9	177.10 (16)
C5—N1—C2—C3	−2.1 (3)	C6—N3—C17—C16	−179.21 (19)
Mn1—N1—C2—C3	174.40 (17)	Mn1—N3—C17—C16	−4.1 (2)
C5—N1—C2—C1	176.54 (18)	C8—C9—C17—N3	−1.4 (3)
Mn1—N1—C2—C1	−7.0 (2)	C10—C9—C17—N3	178.1 (2)
O2—C1—C2—C3	1.8 (3)	C8—C9—C17—C16	179.9 (2)
O1—C1—C2—C3	179.4 (2)	C10—C9—C17—C16	−0.6 (3)
O2—C1—C2—N1	−176.8 (2)	N4—C16—C17—N3	1.8 (3)
O1—C1—C2—N1	0.7 (3)	C12—C16—C17—N3	−178.55 (19)
N1—C2—C3—C4	1.2 (3)	N4—C16—C17—C9	−179.33 (19)
C1—C2—C3—C4	−177.38 (19)	C12—C16—C17—C9	0.3 (3)

### Hydrogen-bond geometry (Å, °)

**Table:**

*D*—H···*A*	*D*—H	H···*A*	*D*···*A*	*D*—H···*A*
N2—H2···O4^i^	0.86	1.97	2.831 (2)	177
O5—H5A···O4^ii^	0.85	2.00	2.829 (2)	164
O5—H5B···O3^iii^	0.85	1.95	2.769 (2)	162
O6—H6A···O2^iv^	0.85	1.79	2.627 (2)	170
O6—H6B···O3	0.85	1.92	2.675 (2)	147

Symmetry codes: (i) −*x*+1, −*y*−1, −*z*+2; (ii) −*x*+1, −*y*, −*z*+2; (iii) −*x*, −*y*, −*z*+2; (iv) *x*−1, *y*, *z*.
